# Towards Understanding KSHV Fusion and Entry

**DOI:** 10.3390/v11111073

**Published:** 2019-11-18

**Authors:** Stephen J. Dollery

**Affiliations:** Biological Mimetics Inc., 124 Byte Drive, Frederick, MD 21702, USA; Dollery@BMI-MD.com; Tel.: +1-301-278-2800

**Keywords:** KSHV, virus entry, fusion, glycoprotein B, glycoprotein H, K8.1, tropism, Ephrin Receptor, Integrin, B cell

## Abstract

How viruses enter cells is of critical importance to pathogenesis in the host and for treatment strategies. Over the last several years, the herpesvirus field has made numerous and thoroughly fascinating discoveries about the entry of alpha-, beta-, and gamma-herpesviruses, giving rise to knowledge of entry at the amino acid level and the realization that, in some cases, researchers had overlooked whole sets of molecules essential for entry into critical cell types. Herpesviruses come equipped with multiple envelope glycoproteins which have several roles in many aspects of infection. For herpesvirus entry, it is usual that a collective of glycoproteins is involved in attachment to the cell surface, specific interactions then take place between viral glycoproteins and host cell receptors, and then molecular interactions and triggers occur, ultimately leading to viral envelope fusion with the host cell membrane. The fact that there are multiple cell and virus molecules involved with the build-up to fusion enhances the diversity and specificity of target cell types, the cellular entry pathways the virus commandeers, and the final triggers of fusion. This review will examine discoveries relating to how Kaposi’s sarcoma-associated herpesvirus (KSHV) encounters and binds to critical cell types, how cells internalize the virus, and how the fusion may occur between the viral membrane and the host cell membrane. Particular focus is given to viral glycoproteins and what is known about their mechanisms of action.

## 1. Introduction

Kaposi’s sarcoma-associated herpesvirus (KSHV) is one of 12 known rhadinoviruses, a genus of the gamma-herpesvirus subfamily of herpesviruses [1,2]. The initial characterization methods of herpesviruses rested upon the virus’s tissue tropism, but now classification based upon genomic sequence homology is the rule [3]. The KSHV is more closely related to zoonotic Rhadinoviruses than other human herpesviruses [4]. Of the rhadinoviruses, KSHV is the only virus known to infect humans [5], and when it does, it can cause two major types of disease: endothelial cell neoplasms (Kaposi’s sarcoma, named after the eminent dermatologist Moritz Kaposi who first described the skin tumors [6]); and the lymphoproliferative disorders of primary effusion lymphoma (PEL) and multicentric Castleman’s disease (MCD) [7,8]. Additionally, KSHV is the causative agent of a severe but rare cytokine disorder, KSHV inflammatory cytokine syndrome (KICS) [9], a disease in which symptoms are similar to MCD, but lymphadenopathy is not salient [10]. Although the route of KSHV transmission is not entirely understood, infection is believed to occur primarily through salivary transmission [11,12]. Viral loads have been estimated at up to 50,000 copies per mL of saliva in shedding individuals [13,14].

**Figure 1:**
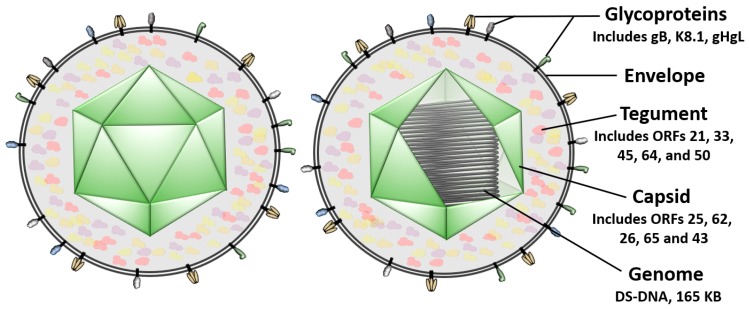
A diagrammatic representation of a Kaposi’s sarcoma-associated herpesvirus (KSHV)virion. Viral glycoproteins, the lipid envelope, tegument, capsid, and double-stranded DNA genome are indicated. On the right, the capsid is depicted with a cut-away section to reveal the double-stranded DNA genome inside. gB: glycoprotein-B; gHgL: glycoprotein-H and glycoprotein-L.

KSHV is a typical herpesvirus (Figure 1); inside the viruses’ icosahedral capsid is a tightly packaged 165-Kb linear double-stranded DNA genome [15,16]. A proteinaceous layer of tegument surrounds the capsid and contains several organized capsid-associated proteins, several loosely-associated proteins, and viral RNAs [17,18,19,20,21]. A host-derived lipid bilayer termed the viral envelope is the last layer that surrounds the whole particle [22]. Viral envelope glycoproteins transverse the viral envelope and are responsible for the initial virus–host interactions [23,24]. Viral envelope glycoproteins K8.1A, glycoprotein-B (gB), and the heterodimer of glycoprotein- H and glycoprotein-L (gHgL) are widely regarded as the most important for virus entry and are the best understood of the KSHV glycoproteins.

Recent discoveries have uncovered new receptors for gH in addition to those known for gB. K8.1A has also been shown to be critical for infection of at least some B-cells. Structurally, endodomain regions of the glycoproteins reside within the virion, and transmembrane-regions bridge through the lipid bilayer connecting to the ectodomain region. Glycoprotein ectodomains protrude outward from the virion and are often depicted as spikes or studs, giving the virion a sea mine-like appearance.

## 2. KSHV Entry

The KSHV envelope glycoproteins can be categorized into two groups: a group of KSHV-specific glycoproteins and a group in which members are homologous to other herpesvirus glycoproteins. The KSHV-specific glycoproteins found in the envelope are K8.1A, ORF4, ORF28, ORF45, and ORF68 [19,20,25,26]. Envelope glycoproteins with homologs in other herpesviruses are gB, gHgL, glycoprotein M, and glycoprotein N, and are correspondingly named after their Herpesviridae forerunners [27,28,29,30,31]. In terms of KSHV entry, gB and gHgL are the best characterized to date, perhaps in part due to their known importance in other herpesviruses and subsequent discoveries that have corroborated their importance to KSHV. The functions of several glycoproteins in the virion have yet to be elucidated, but it is speculated that the glycoproteins that remain largely uncharacterized have regulatory functions or functions that are important in vivo that are undetectable by in vitro systems.

**Figure 2:**
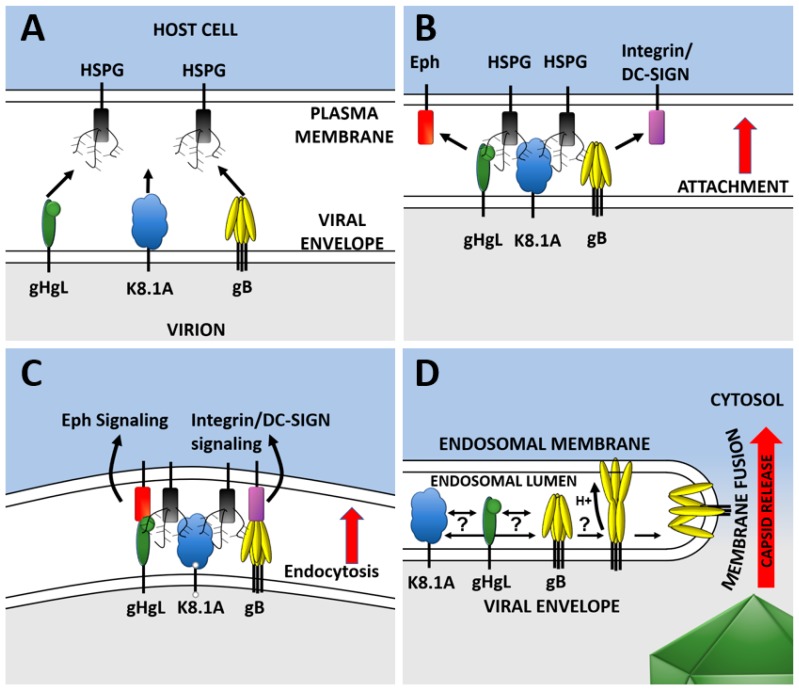
Steps of KSHV entry leading to capsid release. (**A**) KSHV glycoproteins in the virion and the host cell come into close proximity. (**B**) Multiple KSHV glycoproteins bind to heparan sulfate proteoglycans (HSPGs) containing molecules on the host cell plasma membrane. (**C**) Glycoproteins are now close enough that they specifically interact with receptors/signaling receptors on the host. Signaling receptors trigger endocytosis and virions are brought into the cell. (**D**) Signal transduction may occur between receptor binding molecules, intermediaries, and the fusion effector molecules. Fusion occurs once the virus reaches a low-pH compartment and may directly or indirectly trigger fusion glycoproteins. The capsid (green) is released into the cell cytosol.

KSHV entry is a sequential, multistep process. KSHV glycoproteins initially attach to target cells in a nonspecific manner. Multiple interactions with cell surface proteoglycans facilitate attachment to the cell surface. Attachment to heparan sulfate proteoglycans (HSPGs) as binding receptors have been shown to occur through gB, gHgL, and K8.1A in a redundant manner (Figure 2) [30,32,33]. HSPG interactions are not strictly essential, but binding greatly enhances entry into cells where HSPGs are present by concentrating virions at the cell surface [30,32,34]. Once virions attach to the cell, viral glycoproteins are in close enough proximity to cell-surface receptor molecules and specific interactions can occur with surface molecules that function as internalization receptors. gB, gHgL, and perhaps more glycoproteins have regions that function as ligands that bind to complementary receptor sites on specific cell surface molecules. The presence or absence of receptors governs entry into that cell type. gHgL and gB are known to bind to a range of ephrin receptors and integrins, respectively (Table 1). 

These specific binding events are known to trigger a range of select endocytic entry pathways dependent on cell type [50,51,52,53,54,55]. Specific receptors are important for infection, but inhibition of specific receptor binding is rarely reported to reduce infection more than 2–3 logs. This may be due to KSHV’s ability to bind multiple internalization receptors through multiple molecules (see Table 1). A relatively large number of studies have identified accessory factors and signaling pathways that KSHV triggers to allow the virus access to the cytoplasm [56]. The endocytic pathways triggered include clathrin- and caveolin-mediated endocytosis, macropinocytosis, and undefined endocytic entry pathways [47,56,57,58]. The common theme appears to be that regardless of cell type and entry pathway, endocytosis results in the virus gaining access to low-pH compartments. Following the initiation of endocytosis, the virus must still overcome the barrier of the endocytic membrane to gain entry into the host cell.

At some point during the binding and internalization process interactions between glycoproteins such as gHgL, K8.1, and gB may be required. These interactions are critical in other herpesviruses; however, the specific triggers and interactions leading to fusion of the viral envelope with the endosomal membrane are not known for KSHV [59,60,61,62]. Membrane fusion is known to occur once KSHV reaches low-pH compartments, indicating that low pH may be a trigger for the KSHV fusion machinery. In other herpes viruses, gB, the driver of fusion, is triggered by a series of increasingly well-defined steps, including low-pH activation [63,64,65,66,67,68]. Regardless of the exact mechanism, KSHV envelope fusion occurs with an endocytic vesicle membrane and allows delivery of the capsid into the cytoplasm. Membrane fusion is over, and the naked capsid is then directed to the nucleus where replication begins. The default mode of replication for KSHV is latency which ordinarily results in lifelong cryptic infection. 

## 3. Host Cell Tropism

**Figure 3:**
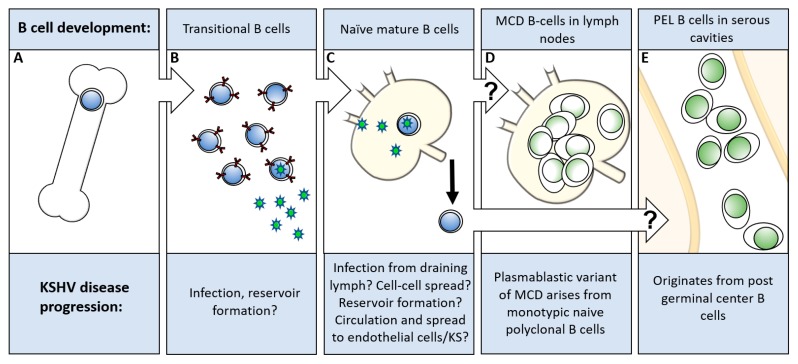
Model of KSHV B cell disorder development. (**A**) Stem cells develop into immature B cells in the bone marrow. (**B**) Transitional B cells expressing Immunoglobulin M-lambda light chain can be found in the bone marrow, peripheral blood or spleen. (**C**) Naïve/mature B cells migrate to the secondary lymphoid organs. (**D**) The development of MCD in the secondary lymphoid organs. (**E**) The development of PEL in body cavities such as the pleural space, pericardium, and peritoneum. PEL: primary effusion lymphoma; MCD: multicentric Castleman’s disease.

When considering virus entry into the host cell, the types of virally infected cells that result in disease are of critical importance. The molecular interactions needed for infection can vary significantly between susceptible cell types. KSHV is believed to be transmitted via a salivary–mucosal route in the majority of cases [69,70]; however, the first cells KSHV infects could be local epithelial cells, endothelial cells, dendritic cells, macrophages, and lymphocytes. Potential routes of dissemination and disease progression in the host are vaguely understood (Figure 3). The origin of the cell types that result in KSHV-related diseases is an open topic of discussion, and much of the current research, while compelling, often highlights that more research is needed. Kaposi’s sarcoma (KS) is routinely thought of as an endothelial cell neoplasm. However, the exact cellular origins of the disorder and how they become infected is still not certain [71,72,73,74]. The primary cell type found in the KS spindle fibers is believed to be of endothelial cell origin based on the observation that there are endothelial cell markers of several types of endothelial cell present [71,72]. The origin of these is nebulous as infected lymphatic endothelial cells have been shown to display lineage markers of blood endothelial cells and vice versa [73,74]. Cells in neoplasms also display markers of dendritic cells, macrophages, and smooth muscle cells [75,76,77,78]. Several studies show that KSHV can infect almost any cell type such as human endothelial, epithelial, keratinocytes, monocyte, macrophage, dendritic cells, T-cells and fibroblast cells, and several types of animal cells [12,79,80,81,82,83]. In general, infection of these cells does not result in drastic differences to the cell. Early in KS development, KS nodes are rich in immune cells, indicating a potential role for immune cells during macule formation. However, the increase in immune cells could be driven by infected cells and not be the source of the virus [84]. It is speculated that infected B cells proximal to endothelial cells may play a role in KS formation. 

Many virus entry studies have focused on endothelial and fibroblast cell types as they appear to be vitally important and are easy to work with respectively. Perplexingly, and in stark contrast to Epstein–Barr virus (EBV), KSHV is almost totally incapable of infecting B cell-lines in culture [32,33,85,86]. B cells are of critical importance to KSHV; they form the reservoir of latent virus in the host, they are the cell type of origin for PEL and MCD and a possible source of KSHV in the saliva [87].

In the cancer field, the phenotype of a transformed B cell is often assumed to mirror its cell type of origin [88]. Cells may, however, be infected and then transformed following differentiation, making it possible that B cells at an earlier stage of development are infected, differentiate, and then result in diseases [89]. KSHV infected B cells are also not generally assumed to undergo cell lineage altering rearrangements upon infection (e.g., from plasmablasts cells to memory B cells), although a recent report has shown that infection may drive alternate light chain expression in (from κ to λ) for reasons that are still unclear [90]. B cells also undergo variable, diversity and joining gene segment (VDJ)recombination and somatic hypermutation events during the different stages of development, making it hard to imagine how a transformed late-stage B cell would undo extremely specific genomic rearrangements and appear like an early stage B cell. The origins of PEL and MCD are believed to occur from different stages of B cell development [91,92,93]. It remains an open question as to which type of B cell or types of B cell precursors are susceptible to KSHV. For MCD and PEL, it is unlikely that activated B cells expressing dendritic cell-specific ICAM-grabbing non-integrin (DC-SIGN) are directly responsible for reservoir formation. It is more likely that a range of B cells, or a B cell type early on in the lineage is critical for infection. The potential range of B cells that may be critical for disease and the difficulty infecting them hinders the study of B cell infection. In addition, the route of viral dissemination in the host is incompletely understood [89].

Interestingly from an entry perspective, KSHV is not ubiquitous in the general population like EBV and other herpesviruses. The lower rates of infection may be a reflection of how difficult it seems to infect the reservoir cell type. If this is the case, therapies that inhibit B cell infection may prove especially useful in prevention. Several reports detail ways to assay infection in B cells [34,36,48,86,94,95,96]. Studies have been performed in tonsillar B cells, activated peripheral blood B cells, and cell lines that are engineered to express factors that enhance general virus entry [48,86]. The recent discovery of the unmodified MC116 B cell line as susceptible has aided the discovery of some apparent differences between B cell infection and other cell types (discussed below) [34,36]. Interestingly, the MC116 cells have phenotypic markers of cells from early on in the B cell lineage and so they may model B cells relevant to reservoir formation [34]. 

The nearly ubiquitous expression of heparan sulfate (HS) is believed to be a significant contributing factor in the ability of KSHV to enter so many cell types. It is now becoming increasingly understood that in addition to attachment, KSHVs ability to specifically interact with large families of molecules as receptors is a major contributing factor to its broad tropism range. Even the notoriously difficult to infect B cells appeared to be somewhat more susceptible when variant cell lines expressing HS were tested [86]. However, the absence of HSPG on the majority of B cells may mean that KSHV has to rely on specific interactions for attachment in those cells. 

## 4. KSHV Entry Glycoproteins

### 4.1. K8.1

The K8.1 gene gives rise to two alternatively spliced reading frames encoding glycoproteins K8.1A and K8.1B [97]. K8.1A is the predominant form incorporated into the viral envelope and is the most abundant envelope glycoprotein [20,98,99,100]. K8.1 is extremely immunogenic in the host [101]. In studies of function, K8.1A was shown to bind HSPG with high affinity [32,102]. However, it was later shown that K8.1A is dispensable for infection of endothelial cells and 293 cells, giving rise to the idea that in the absence of K8.1A other glycoproteins could perform this function [32,103]. K8.1A was recently shown to be critical for entry into a B cell-line and a large subset of tonsillar B cells, but not other cell types, showing that virion K8.1A may be a critical determinant governing the infection of B cells [34]. The requirement for K8.1 was shown to be independent of its HSPG binding activity, indicating that another interaction is essential to the mechanism. The findings are especially interesting when considering other gamma-herpesviruses. Many gammaherpesviruses (rhadinoviruses RV2, MHV-68, BoHV-4, and herpesvirus saimiri, lymphocryptoviruses EBV, rhesus lymphocryptovirus, and CalHV-3) have a gene that a is positional homolog of K8.1, the product of which is present in the virion [104,105,106,107,108,109]. The gene products, gp350/220 of EBV, gp150 of MHV-68, and gp180 of BoHV-4, are involved in mechanisms governing the selective entry and infection of B cells and epithelial cells, supporting the idea that gamma-herpesviruses encode tropism determinants at this region in the genome [108,110,111,112,113,114,115,116].

The mechanistic reason for the requirement of K8.1A during initial B cell infection is still to be reported. However, interaction with a cell surface receptor would have precedence based on other gamma-herpesviruses [117,118], and K8.1 is known to interact with the cell surface in the absence of HSPG interactions [102]. 

In another line of inquiry, a recent study with a gH deletion mutant virus showed that infection of MC116 B cells may not require gHgL [36] This especially interesting given the observation that K8.1A has been shown to be critical for infection of MC116 cells [34]. It may be that K8.1A performs gH-like functions during the infection of B cells. [34,36]

### 4.2. Glycoprotein B

Glycoprotein B (gB) is initially synthesized as 110-kDa protein which can be proteolytically cleaved at a furin cleavage site (aa440–441, RKRR/S) and glycosylated to give two disulfide-linked subunits of 75 and 59 kDa. gB is present in the virion in both full-length and cleaved forms with the cleaved protein being the most prevalent [29,33,119]. However, the level of gB cleavage is potentially cell-type-specific [33], and the function of gB cleavage in KSHV is unknown. Studies with the closely related MuHV-4 show that gB cleavage may affect entry in a cell type-specific manner [120]. This is especially intriguing as early reports of KSHV gB processing reported cleavage occurring in a cell-type-specific manner [29,33]. Thus, gB made in one cell type may more readily infect certain cell types.

gB is abundant in the virion and is heavily *N*-glycosylated at 15 potential *N*-glycosylation sites [29,121]. Functionally, it is one of several glycoproteins known to bind to cell surface HS moieties to facilitate viral attachment to the host cell [119]. Following attachment to HSPG, gB binds to integrins in the host cell membrane. The *N*-terminal ectodomain region has a classic Arg-Gly-Asp (RGD) binding motif, which was initially identified in fibronectin and functions to mediate interactions between fibronectin and integrins during cell adhesion [38,119]. gB can interact with numerous integrins and has been shown to bind to α3β1, αVβ3, and αVβ5 for entry into human fibroblasts and epithelial and endothelial cells (Table 1) [38,39]. Binding integrins is believed to trigger a cascade of cellular signaling pathways that result in virion internalization. The interaction with integrins is well characterized as inducing focal adhesion kinase (FAK), steroid receptor coactivator (Src), Phosphoinositide 3-kinase (PI3K), and Ras homologue GTPase (Rho-GTPase) signaling and bring about endocytosis [43,56]. However, the evidence for which integrins are needed for KSHV entry into particular cell types is occasionally mixed [39,44,57,122,123]. It was reported recently that integrins might not be needed for infection in every cell type, supporting the idea that KSHV can interact with multiple receptors and that, as long as endocytosis is triggered, KSHV can enter the cell [37]. The results of several studies suggest there may be several, as yet unidentified, KSHV receptors. 

DC-SIGN has also been found to be an entry mediator for KSHV into activated B cells, dendritic cells, macrophages, and monocytes [46,48,121]. During glycosylation, gB incorporates high mannose sugars which act as ligands on gB for binding to DC-SIGN [29,121]. DC-SIGN binds gB through a carbohydrate recognition domain (CRD), which is also needed for human immunodeficiency virus (HIV) gp120 binding and intercellular adhesion molecule (ICAM) binding functions [121]. DC-SIGN is of known importance to the pathogenesis of viruses such as flaviviruses and HIV [124,125]. DC-SIGN has been shown to bind several other viruses, including other herpes viruses. However, the significance of the interaction to pathogenesis is not clear [126]. DC-SIGN was the first receptor identified for KSHV infection of B cells. However, as discussed above, these may not be the B cells that directly lead to disease or reservoir formation. DC-SIGN binding may play a role in KSHV spread throughout the host through helping infection spread via non-reservoir cell types.

Based upon sequence homology, gB is likely to be the key fusogen employed during capsid release into the cytoplasm. However, there is little structure-function verification of this in KSHV. In the absence of atomic resolution structures for gB, models were generated in silico using the SWISS-MODEL program [127] and provide some fascinating potential insight into functions of the molecule until a KSHV structure can verify them. The post-fusion structure of EBV gB was used to model KSHV gB [23,128,129]. The model predicts that, like other herpesvirus gB homologs, KSHV gB is comprised of five distinct functional domains and is homologous to type III fusion glycoproteins. The overall structure is described as being reminiscent of a “slouchy and sad stick figure”, due to a globular head region at the top of monomers that bend over relative to an elongated neck region. Multiple contacts are likely to promote trimer formation between protomers and are modeled to have long alpha helices (domain III and V) reminiscent of class I fusion glycoproteins, and beta sheets (domains I and II) reminiscent of class II fusion glycoproteins. Domains I and II form a plextrin-homology domain (membrane/ligand binding) and domain I contains a three-stranded beta-sheet structure with bipartite loops at the tip (fusion loops) that would be predicted to insert into the host cell membrane during fusion [65,130]. Domain IV of gB is predicted to have an enlarged domain IV (globular head) and an extra highly-glycosylated helix (compared to EBV gB) in domain II. The very *N*-terminal region of EBV was too disordered to resolve, and this is the location of KSHVs RGD binding motif. The motif may be expected to be at the top of the post-fusion ectodomain and appears to be highly flexible based upon how disordered the region is in EBV. Both attributes would serve KSHV-gB well, should they be present in the pre-fusion form.

### 4.3. Glycoproteins H and L

KSHV gH is a 120-kDa glycoprotein that binds non-covalently to the 42 kDa glycoprotein gL to form a heterodimer. Like other herpesviruses, the co-expression of gL is vital for efficient processing, incorporation into the virion, and functionality of gH [42,131]. gH and gL have been shown to interact with HSPGs. However, experiments that block this function have shown that KSHV can still bind HSPGs again demonstrating the redundant function of HSPG interactions for KSHV glycoproteins [24,28,35,51,131].

gH is also known to bind to cell surface ephrin receptors and induce internalization into endothelial, epithelial, and some B cells (Table 1) [40,41,42,45,49]. Ephrin receptors are the largest family of receptor tyrosine kinases and are known to play a role in a number of highly diverse cellular functions, often binding and internalizing effector molecules [132]. Studies show that Ephrin receptor A2 (EphA2) binds to gHgL and internalizes KSHV, resulting in the infection of endothelial cells [40]. Experiments with soluble gHgL showed that gHgL had to be crosslinked to trigger the receptor pathways indicating presentation of multiple gHgL molecules in close proximity are needed to activate EphA2 signaling. This is presumably similar enough to the arrangement of gHgL molecules on the virion. In addition to Eph A2, gHgL was also shown to bind to several other ephrin receptors, but the affinity for EphA2 was by far the strongest [41]. The roles of EphA2 in the regulation of micropinocytosis induced by KSHV were also discovered at a similar time [133,134]. More recently it was shown that EphA4 might also function as an alternative ephrin receptor in several cell types [45]. Another recent report suggests from CRISPR knockout cells that both integrins and Ephrin receptor family proteins are so abundantly expressed that they may function redundantly [37]. The absence of integrin subunits made little difference to infection of Caki or HeLa cells, and in the absence of multiple subunits, infection in Caki cells was still seen. The knockout of EphA2 significantly reduced infection, but the defect was rescuable by ephrin receptors other than A2. In cells permissive to infection, the affinity for internalization receptors would arguably dictate which receptor is the predominant trigger of internalization should multiple receptors be present. This may not hold true if that lower affinity binding partners are more efficient at internalization once triggered.

A more detailed analysis of KSHV gHgL has revealed regions in gH that are essential for gL binding and regions that appear essential for EphA2 and EphB3 binding. Point mutations in gH aa47 and aa49 sharply reduced interactions with gL and the absence of gL from the complex reduced interactions with EphA2. Analysis of mutations to regions in gH that did not abolish heterodimerization with gL found a region in gH that is also important for Eph interactions. Mutations in the E-L-E-F-N motif (Glu (50)-Leu (51)-Glu (52)-Phe (53)-Asn (54)) of gH and the surrounding area showed decreased interaction with EphA2 but not gL. In situ analysis modeled on EBV gHgL predicts that the E-L-E-F-N motif in domain I of gH rests near the N-terminal domain of gL and may be structurally supported by gL, thus stabilizing Ehp binding [42]. It is known that EphA2 is not expressed on most B cells [36,42,131], further highlighting that KSHV may use alternative receptors to enter B cells. EphA4 has been shown to be present on MC116 B cells [36]. However, in the same report, it was demonstrated that virions devoid of gH were still able to infect MC116 cells, indicating a novel entry mechanism may be employed in this cell type [36]. This finding may be corroborated by a fusion assay in which EBV gHgL mediated fusion with target B cells but KSHV gHgL did not [135].

A gHgL structural model was also created based on the EBV structures and the model predicts several virus-specific adaptations [23,60,136,137]. gH is predicted to have four domains and binds noncovalently to the smaller gL which is comprised of a single domain. KSHV gH and gL form more extensive interprotomer contacts than EBV gHgL. In EBV, the binding of gp42 results in the widening of a binding pocket that is hypothesized to result in inter-protein binding interactions that result in fusion in B cells. The corresponding regions in the predicted KSHV structure have numerous changes that do not alter the overall domain architecture but are likely of tremendous importance for as yet unknown inter-protein interactions. It will be interesting to see whether a KSHV glycoprotein can substitute for gp42 in some way, activating gHgL during entry. The activation of gHgL in several herpesviruses is critical for the activation of gB in the build-up to fusion. 

### 4.4. Other Virion Glycoproteins

Significantly less is known about the other virion glycoproteins. As with other herpes viruses, gM and gN are present in the KSHV virion. They are thought to be present in a heterodimeric complex with gN serving as a chaperone to gM during processing. The roles and functions of gM and gN in KSHV entry remain primarily uncharacterized. However, they have been shown to regulate fusion negatively [19]. The potential role of ORF68 in the virion is unclear. ORF4 has been shown to interact with HSPG and other cell surface glycans, indicating that it may contribute to entry at the attachment step [35,138].

### 4.5. Cell Associated Virus/Cell-to-Cell Spread

Cell-to-cell spread and transmission via cell-associated virus are potentially vital parts of the virus life cycle that have yet to be fully explored. A cell co-culture assay has been used to successfully infect several B-cell lines and tonsillar B cells that are otherwise hard to infect. Experiments with iSLK.219 producer cells and BJAB target cells have shown that virus requires the presence of EphA7 in BJAB’s in order to be transmitted [49]. In general, the assay is typically performed with iSLK.219 producer cells, an engineered producer cell line known to produce larger than normal amounts of virus [139]. In the host, infection of neighboring cells via cell-associated virus is quite probable but the locations of cells vital to KSHV reservoir formation is yet to be determined. KSHV has been hypothesized to travel to draining lymphoid organs within dendritic cells and monocytes where it can then interact with B and T cells [89]. A cell–cell spread assay in this context may more accurately depict transmission in the host should infectious particles not be draining to the lymph node and infecting cells directly. 

Interestingly, experiments inhibiting cell–cell spread found that a broad spectrum of soluble A-type ephrins inhibited infection of BJABs pointing to potential differences to cell-free infection. It is yet to be determined if that a virological synapse is formed, or if the vast amounts of cell-associated virus are somehow better equipped to trigger virus uptake [41,139]. 

### 4.6. Fusion

Cell-cell fusion experiments have shown that fusion requires gHgL and gB [55,135,140]. In general cell-cell fusion assays do not directly mirror the fusion that occurs during virus entry and are thus viewed as surrogate-assays for fusion. KSHV cell–cell fusion assays are not as robust as the cell-cell fusion assays for other viruses, making it hard to reproduce data above the core requirements needed for fusion [45]. Initial reports differed as to the types of target cells with which effector cells would fuse. As with other herpesviruses, the most apparent reason for the differences in fusion between the assay and virion fusion function would be the absence of other glycoproteins or even tegument proteins that may regulate fusion. There are, however, several more potential differences. Virion glycoproteins are likely to be derived from an internal organellar membrane and do not contain glycoproteins directly derived from the plasma membrane [141,142]. Interestingly, herpesviral glycoproteins are known to adopt distinct antigenic conformations at different sites throughout the cell [143] and herpes viral glycoproteins are known to recycle from the plasma membrane to internal compartments [144]. The KSHV gB and gHgL responsible for cell–cell fusion in a fusion assay could function very differently to the gB and gHgL found in the virion. Treatments of cells with lysosomotropic agents that raise intracellular pH have repeatedly shown the requirement of low pH for KSHV entry [47,50,57,58,145]. The absence of a low-pH environment in cell–cell fusion may alter the triggering mechanisms, leading to fusion at the plasma membrane. All of these differences are difficult to regulate in the current KSHV cell–cell fusion system. 

## 5. Conclusions

We are at a fascinating time in the development of our understanding of KSHV fusion. This review is aimed to present an overview of the knowledge the field has gathered pertaining to how KSHV enters cells and why it may be necessary for understanding disease and spread. It is anticipated that when structures of the essential glycoproteins for fusion become available that this will significantly expand our understanding of both of these areas and propel future work. While a relatively large amount is known about the mechanisms of endocytic entry into the host cell, the KSHV field lags behind the HSV field in terms of delineating fusion-essential interactions between the critical fusion glycoproteins. It may be that there are no requisite interactions and that low pH is the only trigger needed by gB for KSHV fusion, but this too is unreported. New receptors and evidence for even more receptors are mounting, along with evidence that entry mechanisms that do not require all of the “core herpesvirus fusion machinery” may be at play. Differences observed over the importance of particular receptors in particular cell types may be resolved more quickly if collaborative approaches are taken. There are some striking and important differences emerging between the entry mechanisms of KSHV and other herpesviruses including EBV, and many exciting discoveries are still to be made.

## Figures and Tables

**Table 1:** Summary of KSHV glycoprotein interactions with receptors on pertinent cell types. In the left column, several KSHV-susceptible cell types are indicated with corresponding entry receptors indicated adjacently. The top row indicates glycoproteins known to interact with the indicated receptors, which are believed to be important for entry into the cross-referenced cell type. All the listed glycoproteins are known to bind HSPG with the addition of ORF4, which is not listed [33,35]. HSPG is present on the majority of cells with the exception of B cells. * Recent studies show that these interactions may not always be necessary [36,37]. EphA2: Ephrin receptor A2.

	Virion Glycoprotein		
Cell Type	gB	gHgL	K8.1A
Endothelial	α3β1 [38,39], αVβ3 [39], αVβ5 [39]	EphA2 [40,41,42]	
Epithelial	α3β1 [39], αVβ3 [39,43,44], αVβ5 [39] *	EphA2 [40,41,42] EphA4 [45]	
Fibroblasts	α3β1 [38,39], αVβ3 [39], αVβ5 [39]		
Monocytes	DC-SIGN [46,47], α3β1 [47], αVβ3 [47], αVβ5 [47], αVβ1 [47]		
Macrophages	DC-SIGN [46]		
Dendritic cells	DC-SIGN [46]		
B cells	DC-SIGN (activated B cells) [48]	EphA7 in cell–cell spread [49] *	K8.1A needed [34]
